# Competence-Based Pharmacy Education in the University of Helsinki

**DOI:** 10.3390/pharmacy5020029

**Published:** 2017-06-01

**Authors:** Nina Katajavuori, Outi Salminen, Katariina Vuorensola, Helena Huhtala, Pia Vuorela, Jouni Hirvonen

**Affiliations:** Faculty of Pharmacy, University of Helsinki, P.O. Box 56, 00014 Helsinki, Finland; nina.katajavuori@helsinki.fi (K.N.); outi.salminen@helsinki.fi (S.O.); katariina.vuorensola@helsinki.fi (V.K.); helena.huhtala@helsinki.fi (H.H.); pia.vuorela@helsinki.fi (V.P.)

**Keywords:** curriculum, learning outcomes, competency, stakeholders, generic skills

## Abstract

In order to meet the expectations to act as an expert in the health care profession, it is of utmost importance that pharmacy education creates knowledge and skills needed in today’s working life. Thus, the planning of the curriculum should be based on relevant and up-to-date learning outcomes. In the University of Helsinki, a university wide curriculum reform called ‘the Big Wheel’ was launched in 2015. After the reform, the basic degrees of the university are two-cycle (Bachelor–Master) and competence-based, where the learning outcomes form a solid basis for the curriculum goals and implementation. In the Faculty of Pharmacy, this curriculum reform was conducted in two phases during 2012–2016. The construction of the curriculum was based on the most relevant learning outcomes concerning working life via high quality first (Bachelor of Science in Pharmacy) and second (Master of Science in Pharmacy) cycle degree programs. The reform was kicked off by interviewing all the relevant stakeholders: students, teachers, and pharmacists/experts in all the working life sectors of pharmacy. Based on these interviews, the intended learning outcomes of the Pharmacy degree programs were defined including both subject/contents-related and generic skills. The curriculum design was based on the principles of constructive alignment and new structures and methods were applied in order to foster the implementation of the learning outcomes. During the process, it became evident that a competence-based curriculum can be created only in close co-operation with the stakeholders, including teachers and students. Well-structured and facilitated co-operation amongst the teachers enabled the development of many new and innovative teaching practices. The European Union funded PHAR-QA project provided, at the same time, a highly relevant framework to compare the curriculum development in Helsinki against Europe-wide definitions of competences and learning outcomes in pharmacy education.

## 1. Introduction

In order to meet the expectations of an expert in the health care profession, it is of utmost importance that pharmacy education also creates the knowledge and skills needed in working life and to serve society. Pharmacists are in responsible positions within the health care system and therefore high quality, competence-based pharmacy education is needed [1,2,3,4,5].

Competence can be defined as a specialized system of abilities, proficiencies, or skills that are necessary to reach a specific goal. The term also refers to special functional competencies which are needed in a particular area of expertise [6]. In competence-based curriculum, four features are emphasized: focus on outcomes, emphasis on abilities, a reduced emphasis on time-based training and learner centeredness [7]. Thus, a competence-based curriculum in higher education aims at responding to the needs of the working life. In a competence-based curriculum the defined learning outcomes describe what the students are expected to know, understand and/or be able to do after completing a degree or in order to attain a passing grade in a course [8]. The definition of the learning outcomes take into account not only the expertise in the field, but also the knowledge and skills required for employment [9]. Furthermore, the discipline’s latest developments and trends, as well as the changing learning needs and requirements of employers, need to be taken account [10]. 

The defined learning outcomes describe the knowledge, skills, and attitudes thought to be essential for a professional individual in their working life [11]. It is possible to divide the competencies into three categories: (1) Discipline-specific knowledge and skills; (2) Generic knowledge and skills for knowledge work; and (3) Knowledge and skills related to the expert identity (e.g., [10,12]). Carefully defined learning outcomes should aid the students to better understand what kind of knowledge and skills are needed in their profession after graduation and to direct their learning during (and after) their studies, and thus, aid the students to study more effectively and in a deep-level manner [8]. 

Learning outcomes should be defined for the whole study programs in the University, but also for each study-module and individual course within the program. Thus, the defined learning outcomes are more general at the program level, and more specified in the module and course levels ([8] Biggs & Tang 2011). The defined learning outcomes for the program affect both the curriculum design and teaching. The curriculum structure, as well as the teaching methods, should be derived from and linked to the specified learning objectives [2,8,13]. Furthermore, the assessment should be criterion-based and should validly be related to learning outcomes. John Biggs [2,13] coined the term “constructive alignment” to describe this kind of high quality curriculum design. In a constructively aligned curriculum the learning outcomes, course contents, teaching methods, and assessment are aligned and foster students’ deep-level learning. In other words, constructive alignment highlights the importance of applying the defined competencies and learning outcomes to real-life teaching practices throughout the curriculum. 

An extensive education reform, called ‘the Big Wheel’, was launched at the University of Helsinki in 2015. The aim of this reform is to create competence-based curricula with defined learning outcomes for all the study programs in the University in order to equip the students with the most relevant knowledge and skills needed in today’s working life. In addition, the reform aims at producing the most qualified programs, education, and teaching practices throughout the University in order to foster the students’ deep-level learning via the constructive alignment. Each study program (Bachelor and Master) has a degree program director and a steering group to ensure the quality of the programs. The competence-based teaching in the multidisciplinary programs makes it possible for a student to reach the ability to “think big”, to perceive the whole picture and to assess connections in different contexts. There is also an increasing need to develop broadly such skills as critical thinking, information analysis, and communication (see [5,9,10]).

Faculty of Pharmacy, University of Helsinki, offers Bachelor’s, Master’s, and Doctoral Degree programs in pharmacy education. Students complete the Bachelor’s Degree (180 credits) in three years and Master’s Degree (additional 120 credits) in five years. The studies include a compulsory 3 + 3 month work practice in a community and/or hospital pharmacy during their second and third study-years. The majority of the graduating students find a job in community pharmacies, followed by hospital pharmacies, drug industry and research, education, and administration. 

In the Faculty of Pharmacy, the curriculum reform was launched already in 2012, before the Big Wheel reform of the entire University of Helsinki commenced. There was a true need to define the competencies and to create learning outcomes for the Bachelor’s and Master’s programs in Pharmacy. The teachers, students, and employers all pointed out the need to update teaching contents and practices according to the rapidly developing knowledge and practices in various fields of pharmacy profession. These renewals in Helsinki can be directly related to and compared with the process and outcomes of the PHAR-QA project. This article summarizes these developmental activities and is focused on the processes of the reforms as well as on the outcomes. 

## 2. Process of the Curriculum Reform

The learning outcomes for both the Bachelor’s and Master’s degree programs were created during 2012–2016. In addition, contents and practices of teaching were reformed to meet the intended learning outcomes in order to follow the principles of constructive alignment [3,8,13]. The curriculum reform took place in two phases: the first (2012–2014) focused on the Bachelor’s program and the second (2015–2017) on the Master’s program. Both the renewals were conducted by a named team, which consisted of senior lecturers in pharmacy education, a senior lecturer in higher education, and a member of administrative staff. The teams cooperated closely with the Educational Committee of the Faculty and organized several hearings and interviews for all the professors, teachers, and students in the Faculty. 

In the beginning of the reform, the team carefully studied all the relevant information about the recent evaluations and research reports of pharmacy education and study subjects together with feedback from curriculum and course evaluations. In addition, the team benchmarked exemplary educational units on health care and management in order to find out the best practices for conducting the educational reform. Based on this familiarization, specific aims for the curriculum reform were formed: (1) to create the learning outcomes for the Bachelor’s and Master’s programs which would meet the needs of working life; (2) to create a more challenging curriculum and to develop teaching and assessing methods which would foster students’ deep level learning and active work by students; and (3) to increase the flexibility of the curriculum and the amount of optional studies and thereby strengthen the professional orientation and identity of the students. 

In order to define the learning outcomes, the team arranged hearings for all the relevant parties in autumn 2012. The needs of the working life representatives were found out by interviewing a broad sample of stakeholders in the field of pharmacy. For example, community and hospital pharmacies, the pharmaceutical industry, and authorities in the pharmacy sector were interviewed. The team interviewed also faculty teachers in each discipline, pharmacy students, and international staff of the faculty. The interviews were conducted as focus group discussions. In each interview, there were three to nine participants and they lasted for 60–120 min. Furthermore, all the professors of the faculty were interviewed individually in order to hear their visions in more detail and to engage them to the reform. More than 30 interviews were performed with 83 interviewees altogether.

**Figure 1:**
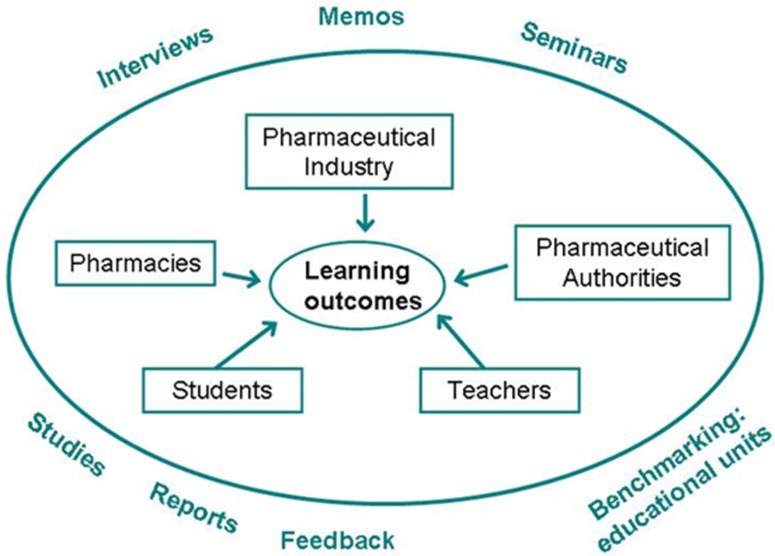
Defining the learning outcomes for Bachelor’s and Master’s programs in pharmacy.

The interviews explored the competencies, knowledge, and skills a pharmacy student should gain by graduation in order to excel in working life in the field of pharmacy. Detailed notes were written during every interview and the notes written were visible to all the participants in the discussions. The notes were commented on and corrected during the discussion if needed. The data of the interviews was analyzed by content analysis method by grouping and categorizing similar themes. In spring 2013, based on the analyses, the team formulated a draft of the learning outcomes including both subject/contents knowledge and generic skills for the Bachelor’s and Master’s programs (see [9,10]). The learning outcomes were further developed, defined, and finalized in several workshops during the spring and autumn 2013. All the interviewed stakeholders, teachers, and students of the faculty were invited to these workshops. The aims of the workshops were to inform about the process and also to discuss important and current themes which rose up from the interviews or from the process. In this respect, the process resembled the iterative character of the Delphi methodology in the PHAR-QA project. Also, the next steps in the reform were decided upon together in the workshops (Figure 1). 

## 3. The Outcomes of the Curriculum Reform 

Formation of the new curriculum was a communal process between the University and working life. The curriculum, its contents, and the learning outcomes for the degree programs were discussed and processed together with teachers, stakeholders, and students (see [3,8]). The atmosphere during the process was enthusiastic and allowed everyone to participate in the process. An increased and systematic co-operation between the university teachers was grounded during the curriculum reform process in order to foster communal learning (e.g., [14,15]). As a result, many of the new practices developed during the curriculum reform were created in longitudinal processes in close co-operation with teachers. In addition, closer co-operation between the university teachers and the stakeholders was established. 

### 3.1. The Learning Outcomes 

The learning outcomes for the programs were defined and, for the first time, the learning outcomes for the programs in pharmacy education in Helsinki also included generic skills (Table 1). When the learning outcomes are defined for the program-level, they are at a more general level. More detailed learning outcomes should be defined at module and course levels of the program [8]. Importance of generic skills in working life were highly emphasized in the interviews. Although the backgrounds of the interviewees were quite different, the learning outcomes proposed by different stakeholders were surprisingly uniform. In addition to critical thinking and problem solving skills, the importance of professionalism rose up in the interviews. Pharmacy students should develop their professional identity during the studies. That includes the importance of realizing one’s role in a health care system and understanding the significance and versatility of the pharmacy field. The core of the pharmaceutical knowledge was, however, to be focused on drug(s) and medication and the education should give a strong basis for this pharmaceutical knowledge and expertise. Defined generic learning outcomes seem to be uniform also at an international level, as shown by Bzowyckyj and Janke [5]. 

The objectives of education leading to the degrees of Bachelor’s and Master‘s of Science (Pharmacy) are: (1) To produce experts for pharmaceutical work in all branches of healthcare and provide the knowledge and skills needed to maintain and improve their expertise; (2) To ensure pharmaceutical expertise, the degrees aim to provide students with the general knowledge and skills described below. Directive 2005/36/EC outlines the knowledge to be acquired through the education leading to the Master’s of Science (Pharmacy) degree.

### 3.2. Curriculum Structures 

**Figure 2:**
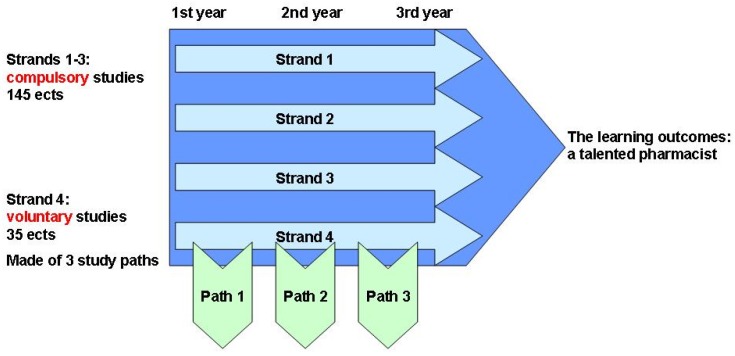
The strand model in Bachelor’s program in the University of Helsinki.

In order to meet the defined learning outcomes and to foster the constructive alignment in teaching, the curriculum structure was modified first. For the Bachelor’s program (the first three study years), a strand model was created by grouping the courses with similar contents to the same strand, to diminish the overlapping of the courses and to promote the smooth continuum of the studies (Figure 2). Coordinators for the four strands in the curriculum, the strand leaders, were nominated in 2014 to lead this process and to develop the constructive alignment and collaboration in the curriculum development and practices. To increase the professional identity of the pharmacy students, the amount of optional studies was increased in the new curriculum. In addition, the optional studies were grouped into three study paths, namely (1) community and hospital pharmacy; (2) industrial pharmacy and pharmaceutical authorities; and (3) research and scientific thinking. 

**Figure 3:**
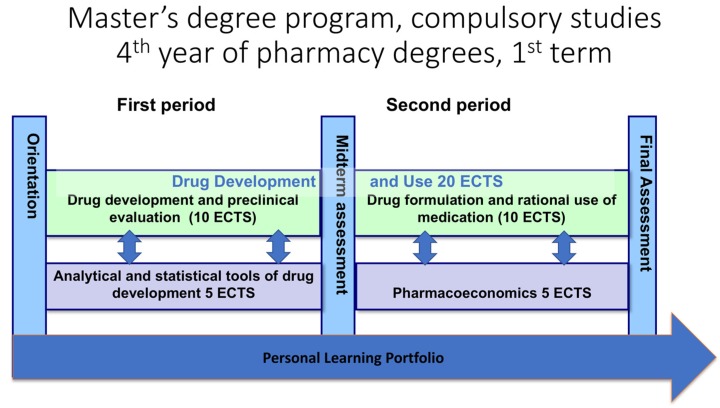
The compulsory studies during the fourth year of Master’s Program in Pharmacy in the University of Helsinki.

During the Master’s program (fourth and fifth study years) the first autumn includes compulsory studies incorporated to one large module, called “Drug Development and Use” (Figure 3). Parallel to this module, there are other compulsory modules including business, economics, analytics and statistics, and also preparation of a personal learning portfolio. The whole term is implemented in close collaboration of all the responsible teachers lead by a named coordinator. In the beginning of the spring term, the students select one specializing study line from seven different disciplines within the field of pharmacy. During these advanced major subject studies the students prepare their Master’s Thesis. The program also contains advanced level optional studies. 

As a result of the Big Wheel reform, steering groups with degree program directors were nominated for the Bachelor’s and Master’s programs in the end of the year 2016. These groups co-operate with each other and lead all the teaching and education development practices from now on, while different working groups, like strand leaders, fall within the guidance of these steering groups. 

### 3.3. Projects Based on the Defined Learning Outcomes during the Pharmacy Education 

A few projects extending over the whole curriculum were established to respond to the requested theoretical and generic learning outcomes, and further, to foster students’ deep-level learning: (1) teachers’ workshops within and between the strands in Bachelor’s program and during the first term of the Master’s program; (2) student group work emphasizing the generic skills; (3) portfolio working; (4) progress testing; and (5) the proof demonstration of knowledge/skills. 

#### 3.3.1. Engagement of the Teachers to Constructive Alignment in Curriculum Design

In order to foster the co-operation in teaching and to develop the teaching and assessment methods, the new strand model and the modules in the programs were discussed and developed in workshops lead by the nominated strand leaders (see [14,16,17]). The new learning outcomes of the programs were implemented and defined also for the strands and individual courses. Several workshops within the strands and modules were carried out including discussions between the teachers about the learning outcomes, constructive alignment, teaching, and assessing methods which would foster students’ deep level learning, challenge-level of the studies and the work-load of the learning tasks.

These workshops created an enthusiastic atmosphere between the teachers. Co-operation and alignment between courses were achieved and teaching and assessment methods were developed. The amount of the lectures was reduced, and new teaching methods like flipped classroom, different assignments, and projects, were introduced to the courses. Assessment methods were also diversified to include also self and peer evaluation, oral assessment, and evaluation of the project works. In addition, the timing of the teaching and assessments were coordinated (see [8]). 

From now on, the nominated steering groups of the programs followed the work of the teachers in the strand groups as well as in the module groups and make sure that the teaching and evaluation of the programs were based on the learning outcomes and followed the principles of constructive alignment.

#### 3.3.2. Fostering the Learning of Generic Skills 

In order to help students to achieve the defined learning outcomes, to encourage their deep-level learning, and to achieve generic skills (Table 1), a systematic and explicit approach was integrated to the theoretical studies facilitating students’ active learning process (see [8,9,10]). 

In the very beginning of their studies, students are divided into small groups. Within these groups, they study together the whole academic year practicing generic skills and solving complicated theoretical problems related to theoretical courses. Four challenging theoretical courses throughout the first study year were selected for this purpose. Each course has a specific theme for the generic skills exercises like group forming, learning methods and scheduling, presentation of results of the assignments, and preparation for an examination. Instructions for the groups are given via the Moodle learning environment during the courses and the groups work independently on assignments without teachers. With proper instructions and well-thought exercises the students are able to work in groups without tutoring and solve the theoretical problems given in the courses. 

Student groups produce materials and memorandums to the Moodle. In these memos, students reflect on their study process and present solutions for the theoretical assignments. The students’ outputs are addressed during the lectures afterwards and the teachers also give feedback of the tasks collectively via the Moodle. 

The group meetings succeeded well and the students felt that the groups are effective in helping to understand the theory and to learn generic skills. On the other hand, some students have had problems in understanding the significance of the group work and allocating time for the meetings. Teachers’ experiences have been positive: students’ study success and group working skills have been significantly improved compared to previous years, and less individual tutoring is needed. In the future, the group assignments will be developed further by selecting the most relevant and closely connected assignments to the theoretical courses. The solid basis for group working is established during the first study year. Different kinds of group assignments continue throughout the study program. In the second study year, learning of the generic skills is highlighted by different kinds of self-evaluations related to the theoretical courses. 

#### 3.3.3. The Learning Portfolio in the Study Programs 

A portfolio is a tool to plan and document ones’ education, work demonstrations and skills. A portfolio can include in-depth reflection of ones’ development and transferable skills. In higher education, portfolios can be used as a tool to evaluate how well the theoretical and generic skills are achieved during the curriculum [18,19]. 

In order to visualize the learning and development of the students, a portfolio for the pharmacy programs was introduced (Table 2). In the portfolio, the student reflects on his/her learning with respect to the learning outcomes twice during the academic year. In addition, the student makes personal plans for studies, reflects on learning skills, completes the progress test (Section 3.3.4.) once a year, and summarizes the overall development during the studies via the demonstration of proof (Section 3.3.5). Also, the student reflects his/her knowledge and skills with regards to future employment. In the Master’s program, the student also writes an application with a motivation letter for the main discipline for their advanced studies. All these assignments and instructions are given via Moodle. 

#### 3.3.4. Progress Testing throughout the Curriculum 

A progress test is a longitudinal educational assessment tool which gives feedback to both the student and the teacher about the development of knowledge during the learning process. The progress test is comprised of multiple choice questions, which assess the substance-specific knowledge, and is administered to all students at the same time at regular intervals throughout the program studies. The differences between students’ knowledge level is shown in the test scores: the further a student has progressed in the curriculum, the higher the score. The results of the progress test provide a longitudinal, repeated assessment of the success on theoretical learning outcomes of the entire curriculum [20,21,22].

In the Faculty of Pharmacy, the strand leaders evaluate the results of the progress test. The idea is based on multidisciplinary questions, which measure a deeper understanding of substance concepts and foster the multidisciplinary nature of the questions. The progress test was launched in spring 2015 and it has now been implemented during the first three years of studies. The test acts as an evaluation tool for the degree program, and the students are actively using the progress test to evaluate their own development. The teachers have already began to see which areas of theoretical studies are well learned and which need brushing up. The degree program directors have gained evidence for the development and improvement of the curriculum. Thus, the progress test works nicely in two ways, reciprocally, to aid both the students and teachers alike. Most importantly, by using the progress test, the degree program directors and the steering groups are able to monitor how well theoretical learning outcomes have been reached during the studies. Preliminary findings suggest that the student learning curve is improving steadily as the studies progress, and it seems that the predetermined learning outcomes can indeed be reached by the end of the studies. 

#### 3.3.5. The Demonstration of Proof 

During the reform process, a practical test called the ‘demonstration of proof’ was created to evaluate both the theoretical and generic skills developed throughout the curriculum, and will be launched for the very first time in spring 2017. The practical test is a two-phase two-day event based on group work. The first phase will include group discussions about the development and strengths of students during their studies and with regards to their employment in the future. In the second phase, the students are given an inspirational stimulus describing a real-life challenge in the field of pharmacy. The students need to create a solution to the challenge, which could be an innovation, a product, a practice, or a procedure, which could be implemented further. The students need to use the theoretical knowledge and generic skills they have learned during their studies and work as a team, just like in real working life. The students will present their solutions to a panel, which consists of teachers and stakeholders. The panel evaluates the students’ presentations and rewards the most innovative and creative solutions. 

The aim of this practical is to summarize the theoretical and generic learning outcomes of the study program. Never before have the learning outcomes been assessed at the end of the studies as a whole. The constructive alignment of the teaching and assessment methods should be implemented not only at the course level, but also in the program level. A final book exam, or even a practical exam, which only measures learning-by-heart type learning, does not answer properly to the question of how learning outcomes have been reached. It is more likely that this type of group activity will demonstrate better the constructive alignment and the achievement of the learning outcomes. The intention is to develop a similar kind of practice for the Master’s program as well. [3,8,13,23]. 

## 4. Conclusions 

During the reform process, it became clear that it is absolutely necessary to involve all the stakeholders including teachers and students when reforming the curriculum. Although the reform process was demanding and time-consuming, it was inspiring at the same time. Even though the process was unforeseeable, the teams could hold the processes together by carefully managing, planning, and changing them in the case of altering circumstances. 

The reform process was able to produce the intended learning outcomes. For the first time in Helsinki, the learning outcomes for the programs in pharmacy included also the generic skills, in addition to the theoretical skills. The learning outcomes enable the curriculum to be built based on constructive alignment and to create the knowledge and skills needed in working life. Increased co-operation with the stakeholders will aid in reaching the intended learning outcomes. 

In the Faculty of Pharmacy, many processes—such as workshops for teachers, organized group work for students, learning portfolios, progress tests, and demonstration of proof—were developed in order to foster student’s deep-level learning, to visualize the development of the students, to evaluate and reach the learning outcomes, to ensure the implementation of the constructive alignment and high quality of the study programs. In order to secure the quality of the programs and to create a sense of community and co-operation between the teachers, it is important to nominate committed responsible persons for the sub-structures of the curriculum. In the Faculty of Pharmacy, the nominated program directors with steering groups and strand leaders follow up and make sure that the programs fulfill the criteria of high quality education. 

The University of Helsinki has participated and kept a keen eye on European level educational development, especially the Pharmine and PHAR-QA projects. Health and patient care orientations and development of generic skills, in addition to subject specific knowledge of drugs, are megatrends that request active follow-up, pedagogic capacity, and active measures to keep pharmacy education as one of the front-runners in University education.

## Figures and Tables

**Table 1:** Learning outcomes for the degrees of Bachelor’s and Master’s of Science (Pharmacy).

**Bachelor’s of Science (Pharmacy)**	
**Learning outcomes concerning knowledge of students who have completed the degree:**	**Learning outcomes concerning generic skills of students who have completed the degree:**
Can apply basic knowledge of the natural sciences and biomedicine in pharmaceutical work	Have developed a professional identity and understand their expert role and duties in healthcare
Have a comprehensive command of pharmacotherapy, from the manufacture of medications to their safe and appropriate use	Are capable of critical thinking, that is, can assess information and apply the results of research in their work
Understand the field of pharmacy as a whole, including employment prospects as well as the role and significance of pharmacy in Finnish and other societies and healthcare systems	Have good problem-solving skills, can tolerate uncertainty, and can acquire information independently
Have the language and communication skills required for expert pharmaceutical work	Understand the necessity of lifelong learning, are motivated to enhance their expertise and can act in a self-directed, creative, ethical, and responsible manner in compliance with the principles of sustainable development
Understand the basic economic principles of business operations and the social functions of healthcare	Can communicate and interact both with customers and in multi-professional groups
**Master’s of Science (Pharmacy)**	
**Learning outcomes concerning knowledge**	**Learning outcomes concerning generic skills**
**Students who have completed the degree have expanded the knowledge and skills acquired through their Bachelor’s of Science (Pharmacy) degree, in addition to which they:**	
Profoundly understand the broad scope of the discipline of pharmacy and have a command of its key phenomena, theories, and concepts	Can work as experts, trainers, and developers in multiprofessional groups in both the pharmaceutical industry and the healthcare sector in Finland and abroad
Have a command of the basics of pharmaceutical development, understand the process of pharmaceutical development, and can apply their knowledge as experts in pharmaceutical development and pharmacotherapy	Have a command of key research methods as well as the research-based work method, can draw scientific conclusions and can produce scientific texts
Have acquired good theoretical competence and methodological knowledge in their specialist area	Have acquired the competences needed for research work in their specialist area as well as the competences for independent work in an international multi-professional research community
Can work in an expert environment in compliance with the principles of expert leadership and have the competence to develop in supervisory positions	Can think critically and analytically and apply research-based knowledge in their work, and have acquired good argumentation and problem-solving skills
Have a command of the basic concepts of business administration and understand the realities of business, particularly from the perspective of pharmaceutical medicine	Understand the potential provided by their expertise in various international environments

**Table 2:** The contents of the student portfolio of the pharmacy programs.

Portfolio of the Pharmacy Programs
Reflection of learning in respect to the learning outcomes
Student’s personal study plans
Reflection of the student’s learning skills
Progress testing
Summarization of the development (years 1–3)
Demonstration of proof
Application for the main discipline with a motivation letter
Summarization of the development (years 4–5)
